# Falls and Fear of Falling among Persons Who Receive Housing Adaptations—Results from a Quasi-Experimental Study in Sweden

**DOI:** 10.3390/healthcare5040066

**Published:** 2017-09-29

**Authors:** Gunilla Carlsson, Maria H. Nilsson, Lisa Ekstam, Carlos Chiatti, Agneta Malmgren Fänge

**Affiliations:** 1 Department of Health Sciences, Lund University, Box 157, SE-221 00 Lund, Sweden; maria_h.nilsson@med.lu.se (M.H.N.); lisa.ekstam@med.lu.se (L.E.); carlos.chiatti@med.lu.se (C.C.); agneta.malmgren_fange@med.lu.se (A.M.F.); 2 Memory Clinic, Skåne University Hospital, SE-214 28 Malmö, Sweden

**Keywords:** active ageing, frailty, home modification, intervention, disability, controlled trial

## Abstract

While health might deteriorate through the ageing and disablement process, the impacts of disability can be reduced by adapting the environment. This study aimed to investigate the effects of applying a standardized research-based strategy to housing adaptation as compared to ordinary practice with respect to falls and fear of falling. Another aim was to investigate the overall effects of housing adaptations on fall-related outcomes over time. In total, 196 clients were included at baseline, with follow-up at 3 and 6 months after the housing adaptation was implemented. The only significant difference between the two approaches was identified with respect to fear of falling at 3 months after the housing adaptation, but not after 6 months. The number of clients reporting actual falls increased over time in both sites, whereas the number of reported near-falls decreased most in the intervention site, but without significant differences. Thus, the patterns of differences between the sites are inconsistent, as are the patterns of change in fall-related outcomes. An overall conclusion is that if the goal is to improve fall-related outcomes, housing adaptation should be complemented with other interventions preventing falls and explicitly address the clients’ activity limitations. In addition, longer follow-up times are necessary.

## 1. Introduction

Falls and fall-related accidents represent a major public health issue, accounting for 70% of all in-hospital treatment due to accidents in Sweden [1]. Among older people, falls can result in long-term disability, dependence on other people in terms of activities of daily life, participation restrictions [2,3,4], and reduced quality of life [5], as well as increased morbidity and mortality [6,7]. Consequently, falls contribute to increased societal costs [8], and considerable gains would be achieved with a decline in the number of falls and falls-related injuries, both for individuals and for society as a whole.

Most often people fall while being active, for example, when taking a walk or simply shifting body weight while doing other activities [9,10]. Large objects, stairs and steps, and surface contamination have been identified amongst the most common risk factors for falling [11]. People with disabilities are at a higher risk of injury from falls than those without a disability [12]. The risks of falling increase with age [9,11] and about one-third of people over the age of 65 fall at least once annually [9]. The proportion of fall-related injuries in and around the home is higher for older people as compared to younger [11]. However, younger persons with disabilities also have an increased risk of falling (see, for example, [13]). In addition to age, the main predictors of injurious falls include gait problems, cognitive impairment, previous history of falls, medication use, and mood disorders such as depression [14,15]. Moreover, psychological consequences of falls, such as fear of falling, are common [16]. Fear of falling is an umbrella term that includes constructs such as fall-related self-efficacy, concerns about falling, and balance confidence. It is more greatly associated with female gender, low physical function, and the use of a walking aid [17] and is most prevalent among prior fallers [17,18]. People experiencing fear of falling tend to avoid doing activities [19] with subsequent risks for activity as well as participation restrictions.

In daily life, person-environment-activity interactions are constantly ongoing and changes in one of the components may impact on the others [20]. Changes in the home environment, for example, by means of a housing adaptation, may improve activity performance, reduce dependence on other people [21,22,23,24], and improve the usability of the home [21,25]. Moreover, housing adaptation can contribute to reducing falls and fall-related disability [9,26], in particular, among people at higher risk of falling [9,24]. Housing adaptations have proved to be successful when forming a part of multicomponent falls reduction programs [23]. A housing adaptation is an individually-tailored intervention aimed at improving independence in the individual’s own home by altering the physical environment, for example, by removing thresholds, installing grab bars, or rebuilding the home [27]. The majority of housing adaptations in Swedish municipalities are assessed, certified, and evaluated by occupational therapists. Most of the individuals receiving housing adaptations are older, with age-related health decline (see, for example, [24,28,29]), however, younger or middle-aged people with acute or progressive diseases or injuries are also among the recipients of the intervention (see, for example, [26,29]).

Systematic approaches to housing adaptation management are used to some extent [30], but the majority of housing adaptations are implemented without making use of current evidence, applying unstructured methodology [31,32]. The Swedish housing adaptation legislation states that the aim is to facilitate an independent lifestyle for those with disability in their own home. No details are provided, however, on how to assess needs and the extent of housing adaptation required, etc. [27]. In addition, research on different approaches for housing adaptations is rare. Accordingly, this study aimed to investigate the effects of applying a standardized research-based strategy to housing adaptation as compared to ordinary practice on fall-related outcomes. The secondary aim was to investigate the overall effects of housing adaptations on fall-related outcomes over time.

## 2. Materials and Methods

### 2.1. Study Design and Study Context

This study is part of a quasi-experimental trial with a non-equivalent control group that had a before–after design [33], called the Research Strategy for Housing Adaptation (ResHA) trial. At the intervention site, occupational therapists applied an intervention that consisted of a standardized research-based strategy for housing adaptation management. At the control site, the occupational therapists worked according to their ordinary practice routines for housing adaptation management. In this trial, we hypothesized that, compared to ordinary practice, applying a research-based strategy on occupational therapy management of housing adaptations, including systematic assessment and evaluation, would positively affect relevant client outcomes.

Three municipalities in the south of Sweden were included based on the number of inhabitants, their geographical dispersion, and their similar organizational structures for housing adaptation administration. Medium-sized municipalities (approximately 40,000–50,000 inhabitants) were consecutively asked to participate in the study. Because of the project’s complexity, duration and the effort required, the staff members and the management needed to express a sincere interest in participating in the study. In addition, a readiness to change their practices was a prerequisite to become an intervention municipality. Two of the municipalities accepted to become the intervention site and in order to include as many in the control site as in the intervention site, one municipality was asked and accepted to become the control site. Before the study started in 2013, there was a variation in the number of accepted housing adaptation errands granted in the three municipalities (between 3.4–10.5 per 1000 inhabitants, i.e., around 137–446 per year in each municipality) [34].

Housing adaptation was defined according Swedish legislation [27], signifying that adaptation can be provided for those who experience declining functional capacity in order to eliminate physical environmental barriers in the home, thus promoting independence and safety. Housing adaptation is a publicly-funded intervention in Sweden, administered by a given municipality in response to a person’s application. The full costs of the housing adaptations are granted based on needs assessment and certification by a health professional, most often an occupational therapist employed by the municipality [34].

All non-institutionalized persons above 20 years of age who applied for a housing adaptation grant (via one of the occupational therapists employed in any of the three municipalities, around 45 occupational therapists) were considered eligible to participate in the study. All municipalities used the same inclusion and exclusion criteria. Exclusion criteria were living in sheltered housing and an inability to communicate or follow instructions in Swedish.

### 2.2. Sample

**Figure 1:**
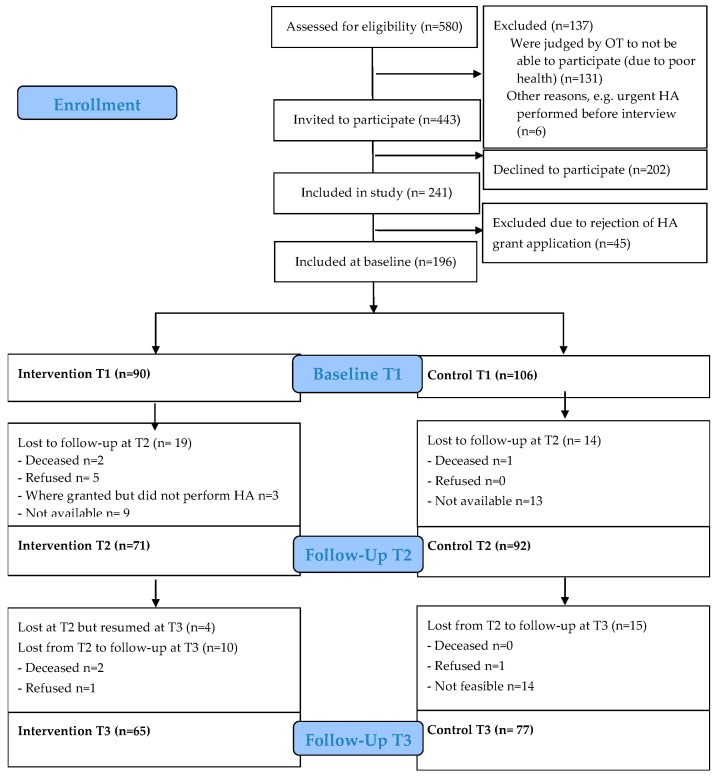
Consort flow chart for the trial.

In total, a consecutive sample of 580 persons met the inclusion criteria to participate in the study (Figure 1). Poor health was not an exclusion criterion, but it turned out that 131 persons were judged by the occupational therapist as unable to participate due to poor health. Six additional persons were excluded due to other reasons, for example, the housing adaptation was urgent and performed before the first interview. The remaining 443 individuals were invited to participate, but 202 (46%) declined. Out of the 202 persons, 64% were women. The average age was 80 years and 40% were 85 years or older. In total, 241 persons accepted to participate, but 45 of them had their housing adaptation application rejected. Therefore, the final study sample consisted of 196 clients at baseline (intervention (I): *n* = 90, control (C): *n* = 106), 163 after three months (I: *n* = 71; C: *n* = 92,), and 143 after 6 months (I: *n* = 65; C: *n* = 77).

About two-thirds of the sample were women and 22% were aged 85 years or older (Table 1). The only statistical significant difference between the intervention and control municipalities was in cognitive function. However, more than one-third had missing data in relation to cognitive functioning; this occurred since the clients did not want to participate or did not complete the assessment.

### 2.3. Intervention

In the interventional municipalities, occupational therapists employed by the municipality applied a standardized research-based practice strategy to the housing adaptations. The intervention guided the occupational therapists with standardized procedures for the assessment and evaluation of person-, activity-, and housing-related aspects at home visits (for details, see [33]). Initially, the occupational therapists attended an extensive training course which included how to conduct the structured assessment procedures by the use of standardized instruments. Assessments were performed prior to housing adaptation certification (T1). The occupational therapists also provided the clients with self-administered questionnaires addressing fear of falling, retrospective falls, and participation. Follow-up visits in their home took place 3 (T2) and 6 months (T3) after the housing adaptation was finalized.

In the control municipality, the occupational therapists did not receive any specific training on the strategy used in the intervention municipality. They continued to work according to their ordinary routines for housing adaptation management, i.e., the occupational therapist combined structured and non-structured assessments of the person-, activity-, and housing-related aspects at home visits, telephone interviews, and in interviews with relatives, staff, etc., tailored to the specific situation of each client [32]. All the clients who met the inclusion criteria and who agreed to participate in the study were contacted by a project administrator (i.e., a specifically trained occupational therapist), who collected corresponding data at home visits.

Client outcomes were identified using a comprehensive process. That is, outcomes were chosen based on current Swedish legislation governing housing adaptations [27], and the Swedish Planning and Building Act [41], as well as previous research on outcomes of housing adaptations [9,24,31]. In addition, outcomes were selected based on their close link to housing adaptations, i.e., the use of a mobility device.

### 2.4. Assessments

A *history of falls* [16] was assessed by using the dichotomous (yes/no) question: “In the last six months, have you fallen in such a way that your body hit the ground?” If the client responded yes, the client was asked to state the approximate number of falls. A dichotomous (yes/no) question concerned the history of near falls during the past 6 months; a near-fall was defined as “a fall initiated but arrested by support from a wall, railing, other person, etc.” [42]. If the client responded yes, the client was asked to state the approximate number of near-falls.

*Fear of falling* was assessed by means of two different measures. First, the client answered a single dichotomous (yes/no) question: “Are you afraid of falling?” Thereafter, the short version of the Falls Efficacy Scale International (FES-I) [43,44] was used, which addresses concerns about falling. The short FES-I includes seven items (i.e., activities) with the following response categories (scored 1–4): “not at all concerned”, “somewhat concerned”, “fairly concerned”, or “very concerned”. An additional response option was also added in the current study: “unable/unwilling to reply”. The total score ranges from 7 to 28 (higher scores = more concerned about falling).

For descriptive purposes, assessments included dependence in *activities of daily living* (ADLs), participation outside the home, and global cognitive functioning and functional limitations. Dependence in ADLs was assessed according to the ADL staircase [38,39,40], which has nine items and the following response categories: “independent without difficulties”, “independent with difficulties”, “partly dependent”, and “dependent” (0–3). The total score ranges from 0–27.

Two study-specific questions addressed the frequency of *participation outside home*. The response categories were “almost never”, “yearly”, “monthly”, “weekly”, and daily”. For analytical purposes, the variables were reclassified into three categories: “never/yearly”, “monthly”, and “weekly/daily”.

As a global assessment for *cognitive functioning* the Montreal Cognitive Assessment (MoCA) was used [36,37]. The maximum score is 30 points (1 point is added if the person has 12 years of education or less, up to a maximum total score of 30), and 26 points and more indicates normal cognitive function.

To assess the number of *functional limitations and use of mobility devices*, the personal component sub-scale from the Housing Enabler [35] was used. The presence of twelve functional limitations (i.e., difficulty in interpreting information, visual impairment, blindness, loss of hearing, poor balance, lack of coordination, limitation of stamina, difficulty in moving the head, reduced upper extremity function, reduced fine motor skill, loss of upper extremity function, reduced spine and/or lower extremity function) and the use of walking aids or wheelchair were dichotomously assessed (yes/no). The two questions on dependence on mobility device were supplemented by a question on whether they used their mobility devices outside the home, in the entrance, or inside the home.

### 2.5. Data Analysis

We first used both descriptive and inferential statistics to evaluate whether the sample differed significantly in the baseline socioeconomic and clinical characteristics between the intervention and the control sites. Counts and proportions were analyzed using chi-squared and Fisher’s exact test where appropriate. Continuous variables were analyzed using Analyses of Variance (ANOVA) tests.

Data from the two follow-ups after 3 and 6 months was then analyzed. The time frame in the questions about history of falls and near-falls was the last 6 months, which means that 6 months had passed between T1 and T3, but there might be an overlap in reported results between T1 and T2 as well as between T2 and T3. The T2 data for these two questions were therefore not included in the analyses. For the analysis of the follow-ups, we aimed to evaluate differences between the two groups in the main fall-related outcomes. We investigated both differences between actual values and changes in the outcomes at the two follow-ups. We used chi-squared tests for differences at the different time points in proportions and ANOVA regressions for continuous variables. In addition, to evaluate the statistical significance of observed changes in proportions over time, we used the Stata module ptrend for analysis of proportions trends. Last, mixed-linear models were used for obtaining adjusted estimates of changes in the FES-I scores at different time points, adjusting for time of the assessment and FES-I baseline values. All analyses were done using STATA 14.0. For all tests, a statistical level of significance of 0.01 was defined in order to account for the small dimension of the sample and avoid the risk of false-positive results.

### 2.6. Ethical Considerations

After a client had contacted an occupational therapist regarding a housing adaptation errand, the occupational therapist in the intervention as well as in the control municipality asked the client whether he or she was willing to participate in the study. Participation was completely voluntary and after the clients had received oral and written information about the study, they provided written informed consent. If the person was cohabiting, the partner also received oral and written information about the study. Withdrawal or declined participation in the study did not affect further services. The study was approved by the Regional Ethical Review Board in Lund (2012/566).

## 3. Results

The final sample comprised 196 clients who actually received a housing adaptation in the study municipalities: 90 in the two intervention sites and 106 in the control site. Sixty-three percent of the sample were women, and less than 15% of them were below 65 years of age. The majority of the sample lived alone (58%). The sample does not differ significantly between the two areas in terms of demographic, social, and clinical characteristics, except for their dependency on wheelchair use inside the home and for cognitive impairment level. With respect to the former characteristics, the significant difference between the two groups are mainly connected to the higher number of missing values among the sample in the control site (Table 1).

At baseline (T1), slightly more than half of the clients (53.6%) reported that they had fallen during the last six months (Table 2). Roughly the same proportion of fallers can be found among the control and intervention municipalities. Six months after the housing adaptation (T3), the proportion of fallers increased up to 71.8% at the control municipality, whereas the proportion of fallers increased up to 55.4% in the intervention municipalities (*p* = 0.041). After six months (T3), the reported mean number of falls decreased from 2.2 (SD 5.5) down to 1.7 (SD 8.8), with a clearer drop among clients in the intervention group (here the mean changed from 2.4 ± 5.5 to 1.4 ± 3.4). Significant differences cannot be found between the two groups in terms of near-falls. At baseline, approximately 60% of the persons reported near-falls in both sites. With respect to the proportion of those reporting being afraid to fall, while no differences were observed at baseline, self-reported fear of falling was more prevalent at T3 among those in the intervention group as compared to the control group (82.8 vs. 66.7; *p* = 0.001). Coherently with this, fear of falling measured using the FES-I was higher both at baseline (18.2 ± 5.7 vs. 14.1 ± 5.3; *p* < 0.001), T2 (14.9 ± 5 vs. 13.2 ± 4.2; *p* = 0.036), and T3 (16.1 ± 4.9 vs. 13.2 ± 4.7; *p* < 0.001) among clients in the intervention group.

With respect to the longitudinal changes in the fall-related variables, the analyses showed (Table 3) that the proportion of fallers increased by 10.8% between T1 and T3 (test for trend significance: *p* = 0.0473), with the larger increase observed among clients in the control group (18%; *p* = 0.0131). Conversely, the proportion of near-fallers decreased by 6.2%, with a larger reduction in the intervention group. The percentage of those reporting being afraid of falling decreased on average by 2% at T2 and 4.6% at T3, compared with T1 values, with no significant trends. The level of fear of falling measured using the FES-I, however, showed a marked reduction among the intervention group between T2 and T1 (−3.3 ± 5.1 vs. −0.4 ± 5.3; *p* = 0.005) and a substantial stability between T2 and T3. Overall, fear of falling decreased in the sample by 1.1 ± 5.5, but no significant differences were found at T3 between the two groups.

At a multivariate level, when correcting for FES-I baseline value and time, the FES-I scores were slightly reduced at the intervention sites after three months, i.e., 2.1-points reduced mean score. There was no longer a statistical significant difference between the sites after six months (T3) (Table 4).

## 4. Discussion

This study investigated whether a standardized research-based strategy to housing adaptation management had an effect on falls, near-falls, and fear of falling as compared to ordinary housing adaptation practice. The only significant difference between the two approaches could be identified for fear of falling at three months after the housing adaptation, but not in a 6-month perspective (Table 3 and Table 4). This study also aimed to investigate the overall effects of housing adaptations on fall-related outcomes over time. The number of clients reporting actual falls increased over time in both sites, most in the control municipality (18%), whereas the number of reported near-falls decreased most in the interventional municipalities (10.9%), but without significant differences between the two sites. That is, the patterns of differences between the intervention and control sites are inconsistent, as is the pattern of change in the fall-related outcome variables.

There are several plausible explanations to these inconsistent patterns. It might be that, even if the differences in characteristics between the groups were not significant, the clients in the intervention group used wheelchairs to a larger extent both indoors and outdoors already at baseline and they were also younger (Table 1). Given the fact that balance and mobility problems account for falls and risks of falling [45], the number of falls and near-falls is presumably lower among people using a stable mobility device such as a wheelchair compared to people using walking devices such as sticks or rollators, which were more common in the control group.

Another potential explanation for our findings may relate to our sample. We included clients eligible for housing adaptations, however, a history of falls and/or fear of falling were not inclusion criteria, and thus the study may be underpowered for detecting differences. Neither the housing adaptations were specifically focusing on reducing falls or fear of falling. Both the intervention and control groups received a housing adaptation and the goal of those usually involves a broader intention than solely fall-related outcomes, i.e., the clients should be able to live independently in their own home [27]. However, the housing adaptation might not correspond to the location and activity where the individual’s falls occur, or where the circumstances that induce fear of falling are. Although the most common adaptations are related to mobility difficulties, such as removal of thresholds or installation of grab bars [34], other interventions are found that might not have a primary effect on falls, such as storage of mobility devices. In retrospect, it would probably have been advantageous to relate the dichotomous question of fear of falling to indoors and outdoors specifically.

Comparing standardized interventions with ordinary practice is a challenge, since components included in the standardized intervention may also to various extents be included in ordinary practice [46]. For example, the same kinds of assessments as applied in the standardized procedure may be included in ordinary practice as well. In this study, it would mean that there is a potential overlap between the intervention and control groups when it comes to assessments and follow-ups, thus potentially impacting on the outcomes.

The intervention in this study concerned occupational therapy practice and housing adaptation was just one part of the measures the occupational therapist suggested to enhance independent home living, due to additional client needs. A challenge with such an intervention aiming to affect client outcomes is the issue of the degree to which the occupational therapists in the intervention group used the results of the baseline assessments. Did they use the assessments in their housing adaptation decision process, or was the standardized research-based strategy in fact seen as something extra, of little relevance for practice? There is also the question of to what extent the occupational therapy team learnt something from the use of the strategy that they actually used or implemented in their ordinary practice, but these issues were not the focus of this paper. The provision of housing adaptations can be considered a complex intervention, since there are several interacting components, and among other issues, the length and complexity of causal chains linking intervention to outcome vary [46]. Even if it is difficult to standardize the design and delivery of complex interventions, this project was an attempt in that direction [33]. As acknowledged by the Medical Research Council (MRC) framework [46], the context in terms of organizational structure and climate has an impact on how research is used in practice, but also, as in this study, staff turnover might impact on how the occupational therapist as a team adopt the intervention. A follow-up period of 6 months might have been too short to evaluate the effects of the intervention on fall-related outcomes, since many fall-prevention interventions require follow-ups for over a year or longer because of delayed effects [16]. Also, the data collection of falls and near-falls were based on retrospective recall, which may result in underreporting of falls [47], however, this ought not to differ between the intervention and control municipalities. According to recommendations, one should use prospective daily recording as well as additional telephone or face-to-face interviews to rectify missing data [16]. Extensive logistics are, however, required to collect this type of data among the persons belonging to the target group of this study, and the clients might already feel stressed by the housing adaptation per se and by participating in the study. In order to reduce falls, multicomponent interventions seem to have an effect [23]. Besides environmental modifications, multicomponent interventions include exercise, and treatment of underlying diseases and impairments, etc. [9]. Fear of falling was also common (77.8%) in our sample and previous research has shown that the prevalence of fear of falling ranges from 21% to as much as 85% among community-dwelling older adults [48]. In order to reduce fear of falling just like to reduce falls, exercise and/or multifactorial programs are required [49,50]. Besides assessments that explicitly address the clients’ limitations in activity performance, our findings thus suggest that not only falls but also fear of falling should be acknowledged in clinical practice, and in future research addressing clients eligible for housing adaptations. Moreover, our findings further strengthen the support for complementing housing adaptations with other interventions, such as exercise or multifactorial programs in order to affect fall-related outcomes.

A common characteristic among housing adaptation clients is that their functional capacity is too low to overcome environmental barriers in the home in a safe way [51], but on the other hand, as persons in general, they differ in needs and interests. The fact that a housing adaptation might comprise several measurements and is not a uniform intervention should be kept in mind. The content and the magnitude differ, even if most of them result in limited adaptations such as threshold removal or installation of grab bars [34]. More detailed analyses of the specific interventions might also increase the knowledge about the clinical relevance.

### Additional Methodological Considerations

In considering the limitations of this study, we have to mention the possibility of a selection bias, which might affect the generalizability of our results. The oldest population was less likely to participate in the study, potentially due to their frailer conditions. At the end, the participants over 85 years were sufficiently represented in our sample, however, caution should be exercised in this respect. Furthermore, the missing data of FES-I and MoCA (Table 1 and Table 2) need to be reflected upon. We cannot exclude the possibility that some of the missing data of FES-I are in fact due to our amendment of an additional response option (i.e., “unable/unwilling to reply”), which was done in order to determine whether all questions were administered. In the short FES-I, the following is stated: “If you currently don’t do the activity, please answer to show whether you think you would be concerned about falling IF you did the activity”. Our amendment might have overruled this original instruction. The lesson learned is that one should be cautious when adding such a response option, since it might contradict instructions in the scale.

The MoCA may have been lacking for several persons in the control site due to the fact that project administrators, unknown to the clients, made the assessments. Assessments of this kind might be stressful and the clients might not have had the same confidence in the assessors as they have in their ordinary occupational therapist.

## 5. Conclusions

Although this standardized strategy in relation to housing adaptations showed a statistically significant short-term effect on fear of falling, it did not persist 6 months after the housing adaptation. Moreover, there were no statistically significant differences between the intervention and control site in relation to falls or near-falls. An overall conclusion is that if the goal is to affect falls and fall-related outcomes, housing adaptations should be complemented with other interventions preventing falls. Such future intervention studies should also explicitly address the clients’ abilities to perform activities.

## 6. Trial Registration

ClinicalTrials.gov: NCT01960582.

## Figures and Tables

**Table 1:** Characteristics of the sample included in the Research Strategy for Housing Adaptation (ResHA) study.

	T1—Total Sample (*n* = 196)		Intervention Sites (*n* = 90)		Control Site (*n* = 106)		*p*
	*n*	% or mean ± SD	*n*	% or mean ± SD	*n*	% or mean ± SD	
Gender							0.780
– Men	72	37%	34	38%	38	36%	
– Women	124	63%	56	62%	68	64%	
Age							0.314
– ≤64	29	15%	15	17%	14	13%	
– 65–74	37	19%	22	24%	15	14%	
– 75–84	82	42%	34	38%	48	45%	
– ≥85	44	22%	17	19%	27	26%	
– missing	4	2%	2	2%	2	2%	
Living arrangements							0.549
– living alone	113	57%	52	58%	61	58%	
– living with others	82	42%	37	41%	45	42%	
– missing	1	1%	1	1%	0	0%	
Education (in years)	189	9.7 ± 3.2	83	9.7 ± 3.1	106	9.6 ± 3.2	0.775
Dependent on walking aids ^a^							
– outside the home	137	70%	60	67%	77	73%	0.363
– entrance	132	67%	57	63%	75	71%	0.336
– inside the home	122	62%	53	59%	69	65%	0.403
Dependent on wheelchairs ^a^							
– outside the home	63	32%	36	40%	27	25%	0.046
– entrance	43	22%	24	27%	19	18%	0.175
– inside the home	31	16%	21	23%	10	9%	0.015
Social activities outside with others							0.188
– never/yearly	57	29%	26	29%	31	29%	
– monthly	55	28%	29	32%	26	25%	
– weekly or daily	79	40%	31	35%	48	45%	
– missing	5	3%	4	4%	1	1%	
Social activities outside alone							0.113
– never/yearly	85	43%	45	50%	40	38%	
– monthly	19	10%	8	9%	11	10%	
– weekly or daily	85	43%	32	36%	53	50%	
– missing	7	4%	5	5%	2	2%	
Cognitive impairment ^b^							>0.001
– 26–30	48	24%	26	29%	22	21%	
– 18–25	87	44%	50	55%	37	35%	
– 10–17	13	7%	7	8%	6	6%	
– missing	48	25%	7	8%	41	38%	
ADL dependence ^c^	157	12.1 ± 5.8	71	12.2 ± 6.7	86	12.0 ± 4.9	0.554
Number of functional limitations ^a^	196	4.7 ± 1.9	90	4.5 ± 1.9	106	4.9 ± 1.9	0.107

^a^ Measured using the Housing Enabler instrument [35]; ^b^ Measured using the Montreal Cognitive Assessment (MOCA) scale [36,37]; ^c^ Measured using the ADL-staircase [38,39,40].

**Table 2:** Fall-related outcomes at baseline and follow-up at 3 and 6 months.

	T1			T2			T3		
	% or mean ± SD	*p*	*n*	% or mean ± SD	*p*	*n*	% or mean ± SD	*p*	*n*
Fallen in the last 6 months (% Yes)		0.951						0.041	
- Intervention	53.3%		90	-			55.4%		65
- Control	53.8%		106	-			71.8%		78
Total sample	53.6%		196	-			64.3%		143
Number of falls in the last 6 months (mean)		0.493						0.638	
- Intervention	2.4 ± 5.5		90	-			1.4 ± 3.4		65
- Control	1.9 ± 5.4		106	-			2.1 ± 11.5		78
Total sample	2.2 ± 5.5		196	-			1.7 ± 8.8		145
Near falls in the last 6 months (% Yes)		0.801						0.116	
- Intervention	60.9%		87	-			50.0%		60
- Control	59.6%		104	-			57.1%		77
Total sample	60.2%		191	-			54.0%		137
Number of near falls in the last 6 month (mean)		0.536						0.273	
- Intervention	4.7 ± 12.4		90	-			4.4 ± 13.5		78
- Control	3.8 ± 7.8		106	-			2.6 ± 4.7		65
Total sample	4.3 ± 10.2		196	-			3.4 ± 9.7		143
Afraid of falling (% Yes)		0.083			0.267			0.001	
- Intervention	79.8%		84	79.4%		68	82.8%		58
- Control	76.2%		105	71.4%		91	66.7%		78
Total sample	77.8%		189	75.8%		153	73.1%		134
Fear of falling (mean) ^a^		<0.001			0.036			< 0.001	
- Intervention	18.2 ± 5.7		66	14.9 ± 5		52	16.1 ± 4.9		45
- Control	14.1 ± 5.3		87	13.2 ± 4.2		73	13.2 ± 4.7		61
Total sample	15.9 ± 5.8		153	13.9 ± 4.6		125	14.4 ± 5.0		106

^a^ Measured using the Falls Efficacy International (FES-I) scale [43,44].

**Table 3:** Changes in fall-related outcomes between baseline and follow-up at 3 and 6 months.

	∆ T2–T1			∆ T3–T2			∆ T3–T1		
	% or Mean ± SD	*p*	*n*	% or Mean ± SD	*p*	*n*	% or Mean ± SD	*p*	*n*
Fallen in the last 6 months (% Yes)									
- Intervention	-	-	-	-	-	-	2.1%	0.800	65
- Control	-	-	-	-	-	-	18.0%	0.013	78
Total sample	-	-	-	-	-	-	10.8%	0.047	143
Number of falls in the last 6 months (mean)								0.512	
- Intervention	-	-	-	-	-	-	–0.9 ± 4.5		65
- Control	-	-	-	-	-	-	0.1 ± 11.4		78
Total sample	-	-	-	-	-	-	–0.4 ± 9		143
Near falls in the last 6 months (% Yes)									
- Intervention	-	-	-	-	-	-	–10.9%	0.189	60
- Control	-	-	-	-	-	-	–2.5%	0.739	78
Total sample	-	-	-	-	-	-	–6.2%	0.263	137
Number of near falls in the last 6 month (mean)								0.857	
- Intervention	-	-	-	-	-	-	–1.0 ± 19.4		65
- Control	-	-	-	-	-	-	–1.1 ± 7.5		78
Total sample	-	-	-	-	-	-	–0.9 ± 13.8		143
Afraid of falling (% Yes)									
- Intervention	–0.4%	0.958	84	3.3%	0.504	68	3.0%	0.680	58
- Control	–4.8%	0.449	105	–4.8%	0.504	91	–9.5%	0.155	78
Total sample	–2.0%	0.669	189	–2.7%	0.603	153	–4.6%	0.339	134
Fear of falling (mean) ^a^		0.005			0.228			0.094	
- Intervention	–3.3 ± 5.1		43	1.1 ± 4.4		35	–2.4 ± 5.3		35
- Control	–0.4 ± 5.3		68	0.0 ± 3.7		51	–0.4 ± 5.6		56
Total sample	–1.5 ± 5.4		111	0.4 ± 4.0		86	–1.1 ± 5.5		91

^a^ Measured using the Falls Efficacy International (FES-I) [43,44].

**Table 4:** Mixed-linear model-based estimates of change in the FES-I score.

Outcomes (Intervention vs. Control)	Between-Group Difference			*p* value
	Mean	(95% Confidence Interval)		
Assessment				
T1	0.72	–0.38	1.82	0.20
T2	–2.04	–3.35	–0.72	< 0.001
T3	1.42	–0.17	3.01	0.08
